# Favorite Activity Patterns Among Older Adults With and Without Dementia: Findings From the NHATS

**DOI:** 10.1093/geroni/igaa057.939

**Published:** 2020-12-16

**Authors:** Tianxue Hou, Minhui Liu, Christina E Miyawaki, Yuxiao Li, Xiaocao Sun, Siyuan Tang, Sarah Szanton

**Affiliations:** 1 Central South University, Changsha, Hunan, China; 2 Central South University, Baltimore, Maryland, United States; 3 University of Houston, Seattle, Washington, United States; 4 Central South University, Changsha City, Hunan, China; 5 Johns Hopkins University, Baltimore, Maryland, United States

## Abstract

Favorite activities are usually meaningful and valuable to older adults. However, information on favorite activity patterns and their relationship with cognitive function from large samples is still limited. Using Round 1 data from the National Health and Aging Trends Study, we examined favorite activity patterns among community-dwelling older adults with and without dementia (N=6,565). Based on the 8-item Ascertain Dementia (AD8) dementia screening interview, participants were classified into no dementia, possible dementia, and probable dementia. Favorite activity was assessed by asking participants, “What their favorite activity they are currently able to do?” Multinomial logistic regression models were used to examine the association between each of the top three favorite activities and the cognitive impairment categories, controlling for demographics and general health. The sample was on average, 77±7.45 years old, non-Hispanic White (69.8%), female (57.3%), and 35.0% had high school education. The three most popular favorite activities among probable dementia participants were watching TV, walking, and outdoor maintenance. Participants who liked watching TV most were more likely associated with possible dementia (Relative Risk Ratio [RRR] = 1.49, p=0.044) compared to participants without favorite activities. Participants who liked walking most were less associated with possible dementia (RRR=0.58, p=0.003) and probable dementia (RRR=0.39, p<0.001) compared to those without favorite activities. Similarly, participants who liked outdoor maintenance most were less likely to develop possible dementia (RRR=0.48, p<0.001) and probable dementia (RRR=0.27, p<0.001) than participants without favorite activities. Researchers may use older adults’ “active” favorite activities to create tailored interventions to slow dementia progression.

